# Gut Dysbiosis in Chagas Disease. A Possible Link to the Pathogenesis

**DOI:** 10.3389/fcimb.2020.00402

**Published:** 2020-08-19

**Authors:** Marcela de Souza-Basqueira, Roberto Marques Ribeiro, Léa Campos de Oliveira, Carlos Henrique Valente Moreira, Roberta Cristina Ruedas Martins, Diego Castillo Franco, Pâmela Pontes Penas Amado, Marcia Pinto Alves Mayer, Ester Cerdeira Sabino

**Affiliations:** ^1^Departamento de Doenças Infecciosas e Parasitárias, Faculdade de Medicina da Universidade de São Paulo, São Paulo, Brazil; ^2^Instituto de Medicina Tropical da Universidade de São Paulo, São Paulo, Brazil; ^3^Laboratório de Investigação Médica (LIM03), Hospital das Clinicas de São Paulo, Faculdade de Medicina da Universidade de São Paulo, São Paulo, Brazil; ^4^Instituto de Infectologia “Emílio Ribas”, São Paulo, Brazil; ^5^Institute of Environmental Sciences, Jagiellonian University, Krakow, Poland; ^6^Departamento de Microbiologia, Instituto de Ciências Biomédicas, Universidade de São Paulo, São Paulo, Brazil; ^7^Fundação Faculdade de Medicina, Faculdade de Medicina da Universidade de São Paulo, São Paulo, Brazil

**Keywords:** Chagas disease, microbiome, 16S rRNA sequencing, gut, dysbiosis

## Abstract

Chagas disease is caused by the flagellate protozoan *Trypanosoma cruzi*. Cardiomyopathy and damage to gastrointestinal tissue are the main disease manifestations. There are data suggesting that the immune response to *T. cruzi* depends on the intestinal microbiota. We hypothesized that Chagas disease is associated with an altered gut microbiome and that these changes are related to the disease phenotype. The stool microbiome from 104 individuals, 73 with Chagas disease (30 with the cardiac, 11 with the digestive, and 32 with the indeterminate form), and 31 healthy controls was characterized using 16S rRNA amplification and sequencing. The QIIME (Quantitative Insights Into Microbial Ecology) platform was used to analyze the data. Alpha and beta diversity indexes did not indicate differences between the groups. However, the relative abundance of *Verrucomicrobia*, represented primarily by the genus *Akkermansia*, was significantly lower in the Chagas disease groups, especially the cardiac group, compared to the controls. Furthermore, differences in the relative abundances of *Alistipes, Bilophila*, and *Dialister* were observed between the groups. We conclude that *T. cruzi* infection results in changes in the gut microbiome that may play a role in the myocardial and intestinal inflammation seen in Chagas disease.

## Introduction

Chagas disease is an anthropozoonosis found primarily in Central and South America. Six to eight million people are infected, and 65–100 million live in areas at risk of infection (Engels and Savioli, 2006; Schofield et al., 2006; WHO, 2015; PAHO, 2016). The causative agent is the flagellate protozoan *Trypanosoma cruzi*, which in recent decades has been a major public health concern, not only in Latin America but also in non-endemic areas due to migration. A period of 2–4 months following initial infection is referred to as the acute phase and is characterized by high parasite levels in the peripheral blood. This gives way to chronic asymptomatic infection, termed the indeterminate phase. Approximately 30–40% of patients will present with a determinate form (cardiac or digestive) over a period of 20–30 years after the initial infection, while the remaining group does not develop the clinically apparent disease (Rassi et al., 2010).

The pathophysiology of Chagas disease is complex and probably represents an imbalanced immunological response to the parasite, leading to inflammation, oxidative stress, microvascular damage, and fibrosis (Rassi et al., 2010). The severity of myocardial inflammation does not correlate with the number of parasites present in the heart (Teixeira et al., 2006; Girones et al., 2007). This suggests that the immunological response together with the genetic background is key in the development of cardiac disease (Cunha-Neto and Chevillard, 2014).

Previous data suggest that the gut may play a role, not only in the digestive form (megacolon) of Chagas disease but also in the development of cardiomyopathy. Experiments using bioluminescent *T. cruzi* have shown it to circulate from the gut to cardiac tissue (Lewis et al., 2014, 2016). Furthermore, the gut may act as a reservoir of *T. cruzi* in recrudescent infections (Francisco et al., 2015).

The gut microbiome appears to be involved in the host response to parasites. A high abundance of *Lactobacillus* and *Bifidobacterium* in the gut microbiota has been associated with resistance to malaria in animal models. This resistance was transmissible through fecal transplants from malaria-resistant to germ-free mice (Villarino et al., 2016). Moreover, in a murine model of Chagas disease, the gut microbial composition was affected by parasite burden. The concentration of fecal linoleic acid and its derivatives, which can alter the gut immune response, was associated with *T. cruzi* infection, as was the abundance of *Ruminococcaceae* and *Lachnospiraceae* (McCall et al., 2018).

The purpose of this study was to further understand the role of the gut microbiome in the different Chagas disease phenotypes. We evaluated the fecal microbiome in patients with different forms of the disease cardiac, gastrointestinal, and indeterminate and compared the results to healthy controls.

## Materials and Methods

### Study Population and Sample Collection

Patients with Chagas disease and diagnosed with the cardiac (*n* = 30) or indeterminate (*n* = 32) forms were selected from a group of 93 patients previously enrolled in a Brazilian cohort (REDS II—Natural History of Chagas' disease) held between 2008 and 2010 in São Paulo, Brazil (Sabino et al., 2013). Eleven patients with the digestive form (megacolon) under follow-up at the Hospital das Clínicas, University of São Paulo and the Institute of Infectology Emílio Ribas both in São Paulo, Brazil, were also enrolled. Thirty-one *T. cruzi*-seronegative blood donors from the Fundação Pró-Sangue were enrolled as controls. Stool samples were obtained between October 2014 and December 2015. Samples were collected in sterile tubes containing 12 mL of 6 M guanidine HCL/0.2 M EDTA solution. We used a questionnaire to collect demographic variables and information on bowel habits. The exclusion criteria were previous treatment with benznidazole, antibiotic use in the 3 months prior to sample collection, or a body mass index of 40 or over.

### DNA Extraction

Genomic DNA was extracted from 20 g of feces using the Power Soil DNA Isolation Kit® (MoBio Laboratories, Carlsbad, CA) with modifications as described by the Human Microbiome Project (McInnes and Cutting, 2010). The amount and purity of the extracted DNA were determined fluorometrically using the Qubit® BR dsDNA Assay Kit with the aid of a Qubit® Fluorometer 2.0 (Invitrogen Co., Carlsbad, CA).

### Library Preparation and 16S Sequencing

The 16S rRNA V4 variable region was amplified using the 515/806 primer set (Caporaso et al., 2011) with a barcode on the forward primer and sequenced using an Ion Torrent™ Personal Genome Machine (Life Technologies, Waltham, MA) with an Ion 318TM chip kit v2 using 400-base chemistry.

### Data Analysis

Sequences were filtered by length (>280 pb), quality score (*Q* = 30), and minimum expected error (0.1) using the USEARCH tool (Edgar, 2010). Single-end reads were assembled using PEAR software (Zhang et al., 2014). Sequences were clustered at 97% similarity using USEARCH (Edgar, 2010) against the Greengenes 16S reference database (13_8 version) (DeSantis et al., 2006). Operational taxonomic units (OTUs) with singletons (*n* = 1) were removed. Taxonomy was assigned to each OTU by performing BLAST searches against the Greengenes database (13_8 version). Sequences were filtered for bacterial phylotypes for further analyses using the Quantitative Insights into Microbial Ecology (QIIME2) plugin (Bolyen et al., 2019). Alpha-diversity measurements, including species richness, evenness, and Simpson and Shannon's diversity index, were calculated based on the rarefied OTU tables using 37,868 sequences per sample. Differences between taxonomic profiles were identified using the ANCOM test (Mandal et al., 2015) with biom tables collapsed to the genus level as the input file.

Phylogenetic beta-diversity (weighted and unweighted UniFrac) (Lozupone et al., 2011) and non-phylogenetic (Bray–Curtis and Jaccard) methods were calculated using cumulative-sum-scaled (CSS) normalized distance matrices. These matrices were employed to generate ordination plots using principal coordinate analysis (PCoA). All sequencing raw reads have been deposited in the National Center for Biotechnology Information (NCBI) under the project accession number PRJNA471615.

### Statistical Analysis

Variable distributions were assessed graphically and with the Shapiro–Wilk test. Continuous variables were not normally distributed and consequently, and we used non-parametric hypothesis tests such as the Kruskal–Wallis test.

Differences between groups in demographic characteristics and bowel habits were assessed with Kruskal–Wallis and chi-square test, respectively, using the statistical software package GraphPad Prism 6. A *p* < 0.05 was considered statistically significant. The statistical significance of differences in relative abundance between the groups (control, cardiac, indeterminate, and megacolon) at different taxonomic levels was determined using the Kruskal–Wallis and Mann–Whitney U tests in the QIIME v1.8 software package. A *p* < 0.05 after Bonferroni correction was considered statistically significant. The Kruskal–Wallis H test was applied to compare all alpha diversity parameters between groups. PERMANOVA (vegan::adonis) was performed to determine if the separation of sample groups in beta diversity was significant.

## Results

### Participants' Demographics

Participant's demographic and clinical characteristics are shown in Table 1. There was no difference between groups in age, weight, or height. However, the megacolon group was composed only of women, whereas the patients with cardiac and indeterminate forms were primarily men (*p* = 0.0005). The reported bowel habits in the control group differed slightly from the Chagas disease patients, independent of disease phenotype (*p* < 0.0001; Table 1).

**Table 1:** Epidemiological and clinical characteristics of 73 participants with three different Chagas disease phenotypes, cardiac, indeterminate, and megacolon, and 31 controls.

**Characteristics**	**Cardiac (*n* = 30)**	**Indeterminate (*n* = 32)**	**Megacolon (*n* = 11)**	**Control (*n* = 31)**	*p*-value^*^
Age (years), mean ± SD	59 ± 7	58 ± 9	55 ± 11	62 ± 9	0.099
Male sex, n (%)	20 (66%)	22 (73%)	0%	17 (57%)	0.0005
Height (m), mean ± SD	1.64 ± 0.31	1.64 ± 0.08	1.60 ± 0.07	1.66 ± 0.08	0.168
Weight (kg), mean ± SD	71.1± 19.07	72.7 ± 10	66.5 ± 11.5	79.2 ± 22.5	0.321
Body mass index, mean ± SD	27 ± 4	27 ± 3	26 ± 4	29 ± 8	0.549
Bowel habit, n (%)					
Once or more times/day	24 (80%)	29 (91%)	4 (36%)	31 (100%)	
Once every 2 to 3 days	5 (17%)	2 (6%)	4 (36%)	0	<0.0001^**^
Once a week or less	1 (3%)0	1 (3%)	3 (27%)	0	

* *Kruskal–Wallis -test, p < 0.05 as the threshold for significance*.

** *Chi-square test, p < 0.05 as the threshold for significance*.

### Sequencing Metrics

**Figure 1:**
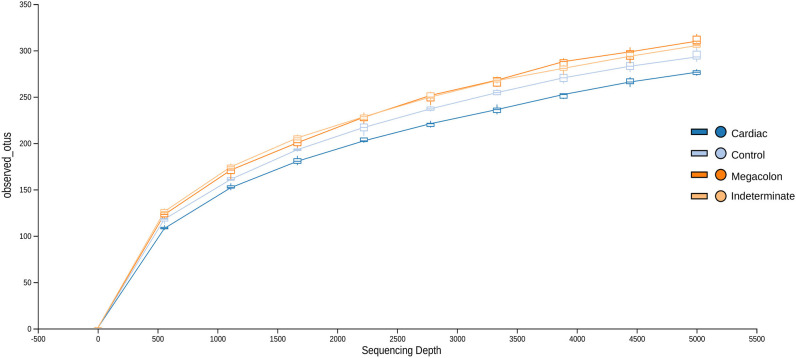
Rarefaction curves demonstrating the estimated number of operational taxonomic units (OTUs) in the microbiome of stool samples of the control group and Chagas disease groups (indeterminate, cardiac, and megacolon phenotypes) as a function of the sequencing effort generated through the QIIME (Quantitative Insights Into Microbial Ecology) platform.

After applying the Torrent Browser software filters, 28,418,519 classifiable sequences, representing 1866 species-level OTUs (median: 1566–1866 OTUs/sample; range: 90–498), were obtained from 104 stool samples. Rarefaction curves showed that the number of observed OTUs plateaued after 37,868 reads (Figure 1). No significant differences were observed in the evaluated alpha diversity indexes (Shannon, Simpson, Chao1, and number of observed species between the groups (Table 2).

**Table 2:** Alpha diversity analyses of the fecal microbiome in Chagas disease phenotypes (indeterminate, cardiac, and megacolon) and controls.

	**Control**	**Indeterminate**	**Cardiac**	**Megacolon**	*p*-value^*^
Chao1 richness estimate	1881.4	1880.0	1809.9	1617.3	0.577
Shannon index	7.1	7.5	7.1	7.2	0.301
Simpson's diversity index	0.973	0.983	0.974	0.980	0.333
Number of observed species	1866	1848	1741	1566	0.751

* *Kruskal–Wallis H-test, p < 0.05 as the threshold for significance*.

Furthermore, the beta diversity index visualized by PCoA of weighted and unweighted UniFrac distance matrices revealed that the microbial composition of stool samples from the controls and Chagas disease patients were not clustered according to their bacterial composition, with *p*-values of 0.110 and 0.103, respectively, according to the Permanova test (Supplementary Figure 1).

### Characterization of the Microbiota of Clinical Forms

Comparing the sequencing results of patients with clinical forms of Chagas disease (cardiac, indeterminate, and megacolon) with the control group, was possible to observe the characterization of important microbiota present in this study population.

The microbiota composition included three predominant genus: *Bacteroides, Prevotella*, and *Faecalibacterium*. The most abundant taxa was *Bacteroides* in control (25.96%), indeterminate (14.30%), and megacolon (19.04%), respectively. The cardiac group was dominated by *Prevotella* (20.15%) (Supplementary Table 2).

**Figure 2:**
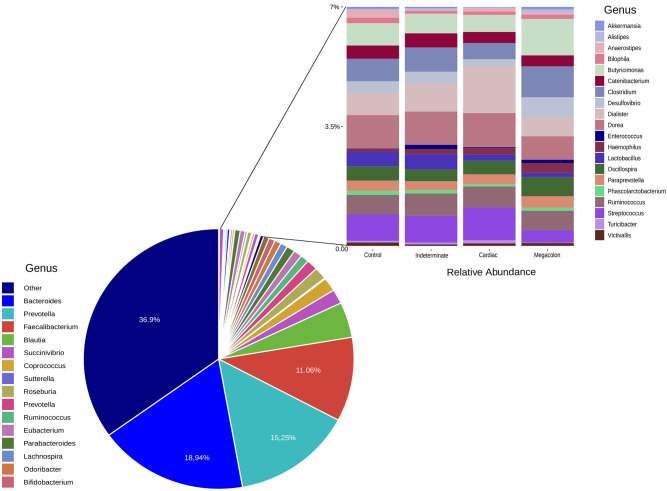
Genera relative abundance (%) based plots. The left plot represents the average relative abundance detected in the stool samples of all the groups (control, indeterminate, cardiac, and megacolon) and the right least abundant genus (<7%).

At the phylum level, the only difference between the groups was in the abundance of *Verrucomicrobia*, although it was only present at a low proportion (<0.1%). This phylum was more abundant in the healthy controls than in patients with the cardiac form (*p* = 0.045) (Table 3). The ANCOM test confirmed these results, demonstrating significant differences between groups at every taxonomic level. This was particularly marked for the *Akkermansia* genus, which was less abundant in the fecal microbiomes of the cardiac phenotype group than in the healthy controls. Figure 2 shows the fecal bacterial abundances at the genus level for the three Chagas disease groups and control subjects (also see Table 4).

**Table 3:** Abundance of organisms in the fecal microbiome of the cardiac phenotype of Chagas disease and control group.

		Control ^*^ (%)	Cardiac ^*^ (%)	**FDR_P**	***p*-value^¬^**	***p*-value Bonferroni correction ^¬^**
Phylum	Verrucomicrobia	0.06	0.02	0.045	0.003	0.045
Class	*Verrucomicrobia*	0.05	0.01	0.062	0.002	0.062
Family	*Verrucomicrobiaceae*	0.05	0.01	0.188	0.002	0.188
Genus	*Akkermansia*	0.05	0.01	0.477	0.003	0.572

* *Data are expressed as means*.

¬ *Mann–Whitney U-test, p < 0.05 were considered significant. FDR_P—false discovery rate*.

**Table 4:** Differences in the fecal microbiome taxonomic profiles in the Chagas disease phenotypes (indeterminate, cardiac, and megacolon) vs. controls.

	**Phylum**	**Class**	**Family**	**Genus**
Control × cardiac^*^	Verrucomicrobia (W 3)	Verrucomicrobia (W 11)	*Verrucomicrobiaceae* (W 31)	*Akkermansia* (W 23)
Control × megacolon^**^	NS	NS	NS	NS
Control × indeterminate^**^	NS	NS	NS	NS

* *Data are expressed as W values using ANCOM test*.

** *NS—no significant differences were observed*.

After Bonferroni correction, the comparisons of relative abundances at the other taxonomic levels did not reach statistical significance. However, some differences, albeit not statistically significant, are important to note. At the class level, *Alphaproteobacteria* and *Betaproteobacteria* were less abundant in the cardiac and megacolon groups (*Alphaproteobacteria*: 0.02, 0.13, and 0.53%; *Betaproteobacteria:* 1.53, 1.21, and 2.27%, in the cardiac, megacolon, and control groups, respectively). The relative abundances of the bacteria that differed between groups at the family and genus levels are shown in Supplementary Tables 1, 2, respectively.

Patients with Chagas megacolon had higher relative abundances of *Rikenellaceae* and *Odoribacteraceae* than the other groups. The abundance of *Rikenellaceae* was 0.39, 0.87, 0.82, and 0.31% in the cardiac, megacolon, indeterminate, and control samples, respectively, while the abundance of *Odoribacteraceae* was 0.99, 1.65, 1.18, and 1.48%. There were significant differences in the relative abundances of members of *Rikenellaceae* (genus *Alistipes*: 0.07, 0.02, 0.04, and 0.02%), *Desulfovibrionaceae* (genus *Bilophila*: 0.11, 0.07, 0.07, and 0.11%), and *Veillonellaceae* (genus *Dialister*: 0.32, 1.02, 0.53, and 0.50%) among the megacolon, cardiac, indeterminate, and control groups (Kruskal–Wallis, *p* < 0.05).

## Discussion

Chagas disease has heterogeneous clinical manifestations, including gastrointestinal disease. The relationship between the resident microbiota and Chagas disease phenotypes is still poorly understood. This is the first study that has sought to describe the fecal microbiome of adult patients with the various Chagas disease phenotypes using next-generation sequencing technology.

Robello et al. (2019) observed a difference in the microbiota between children infected with *T. cruzi* and controls. In the study, children aged 5–15 years with Chagas disease had higher fecal Firmicutes (*Streptococcus, Roseburia, Butyrivibrio*, and *Blautia*), and lower *Bacteroides*. After being treated with benznidazole, the fecal microbiota was normalized in comparison with control children (Robello et al., 2019).

We have shown that the diversity of the gut microbiome in patients with the cardiac form of Chagas disease differed from the other Chagas phenotypes and the control group. These differences were mostly due to a lower abundance of the phylum Verrucomicrobia in the Chagas disease groups. In particular, there was a lower abundance of the genus *Akkermansia*, especially among cardiac patients.

The abundance of *Akkermansia*, a butyrate-producing bacteria, has been associated with a healthy gut and decreased inflammation in several animals (Everard et al., 2013; Li et al., 2019) and clinical (Collaboration, 2016) studies. Interestingly, the use of prebiotics that increases the abundance of *Akkermansia* in the gut has been associated with reduced endotoxemia and insulin resistance (Everard et al., 2013). Increased abundance of *Akkermansia* has been linked with the beneficial effects of a low-protein/high-carbohydrate diet for maintaining gut health (Li et al., 2019). This microorganism has also been associated with a positive clinical response to therapy in patients with cancer. In addition, as a probiotic in mice with unfavorable microbiota, it induces the production of interleukin (IL)-12, favoring an antitumor response (Routy et al., 2018). Interestingly, IL-12 is also involved in the immune response to *T. cruzi* (Marin-Neto et al., 2007), and our data support a role for *Akkermansia* in maintaining a healthy gut.

We have also demonstrated differences in the proportion of a number of bacterial groups when comparing *T. cruzi*-infected subjects to non-infected individuals. However, these findings were not significant after Bonferroni correction. Notably, the cardiac phenotype of Chagas disease was associated with fecal enrichment of *Alistipes* and *Bilophila*, when compared to the other Chagas disease groups. Both *Alistipes* and *Bilophila* are bile-tolerant microorganisms associated with an animal-based diet in humans (David et al., 2014; Wan et al., 2019). *Alistipes* has previously been associated with other diseases, such as diabetes (Qin et al., 2012), colon cancer (Fenner et al., 2007), and gut inflammation (Saulnier et al., 2011). Therefore, future studies should address the role of diet and these genera in gut dysbiosis in the different phenotypes of Chagas disease.

The abundance of *Dialister*, a genus of the *Veillonellaceae* family, was increased in the cardiac patients compared to controls and the other Chagas disease groups. Increased *Dialister* abundance in the gut microbiome has been previously associated with diabetes (Ciubotaru et al., 2015) and its complications (Sanchez-Alcoholado et al., 2017) as well as with inflammatory bowel syndromes (Lopetuso et al., 2018), suggesting its pathogenic potential.

Our findings suggest that infection with *T. cruzi* results in a shift in the gut microbiome toward a dysbiotic community, with lower levels of health-associated microorganisms such as *Akkermansia*, especially in the cardiac form of the disease. These data should be interpreted considering the limitations of this cross-sectional study. Comparisons with other work are limited due to a lack of standardized protocols among different studies of the gut microbiome (Costea et al., 2017; Panek et al., 2018). Furthermore, the progression of Chagas disease from the indeterminate form to the life-threatening cardiac form has previously been associated with a multitude of factors. Future studies to identify biomarkers of Chagas disease progression should be longitudinal in nature. The relationship between microbial composition and other variables involved with the microbiome and disease evolution should be investigated, including serum inflammatory mediators (Keating et al., 2015; Gomez-Olarte et al., 2019; Medeiros et al., 2019), diet (Nagajyothi et al., 2014), serum vitamin D levels (Oliveira Junior et al., 2019), gene polymorphisms (Reis et al., 2017; Carvalho et al., 2019), and habits such as exercise (Geraix et al., 2007; Alves et al., 2019). Other studies relating the gut parasite *Blastocystis* and microbiome also show that the genus *Akkermansia* plays a role in human gastrointestinal health. In this study, they observed an inverse correlation of the *Blastocystis* subtypes 3 and 4, suggesting differential associations of subtypes with host health (Tito et al., 2019).

The association between altered gut microbiome and the different phenotypes of Chagas disease suggests that it may play a role in the pathogenesis. This would be in addition to other possible multifactorial mechanisms, such as tissue parasite-driven damage, immune response, and microvascular and neuronal disturbances. However, it is unknown whether an already altered gut microbiome predisposes a *T. cruzi*-infected subject to disease progression, or if the established disease alters the gut microbiome. Further studies are needed to determine the mechanisms underlying the association between *T. cruzi* infection and the gut microbiome in Chagas disease. However, our results do suggest that manipulation of the gut microbiome could be an additional strategy for controlling the pathogenic potential of *T. cruzi* in infected subjects.

## Data Availability Statement

The datasets generated for this study can be found in the National Center for Biotechnology Information (NCBI), PRJNA471615.

## Ethics Statement

The studies involving human participants were reviewed and approved by The Internal Review Board of the University of São Paulo (CAPPesq number 1.174.958). The patients/participants provided their written informed consent to participate in this study.

## Author Contributions

Conceptualization: MS-B, MM, and ES. Formal analysis: RR, MS-B, CM, RM, DF, and PA. Funding acquisition: ES. Investigation: MS-B and LO. Methodology: MS-B, RR, LO, CM, and RM. Supervision: MM and ES. Visualization: MS-B, LO, CM, RM, MM, and ES. Writing—original draft: MS-B. Writing—review and editing: All authors read and approved the final manuscript.

## Conflict of Interest

The authors declare that the research was conducted in the absence of any commercial or financial relationships that could be construed as a potential conflict of interest.

## Supplementary Material

Supplementary Figure 1 Beta diversity plots for non-phylogenetic (Bray-Curtis and Jaccard) and phylogenetic (weighted and unweighted UniFrac) methods in control and the Chagas disease phenotypes (indeterminate, cardiac and megacolon).
