# Supplementary figures and images for: Gut Dysbiosis in Chagas Disease. A Possible Link to the Pathogenesis
Source: Front Cell Infect Microbiol. 2020 Aug 19;10:402. doi: 10.3389/fcimb.2020.00402 (PMC7466656; doi:10.3389/fcimb.2020.00402)

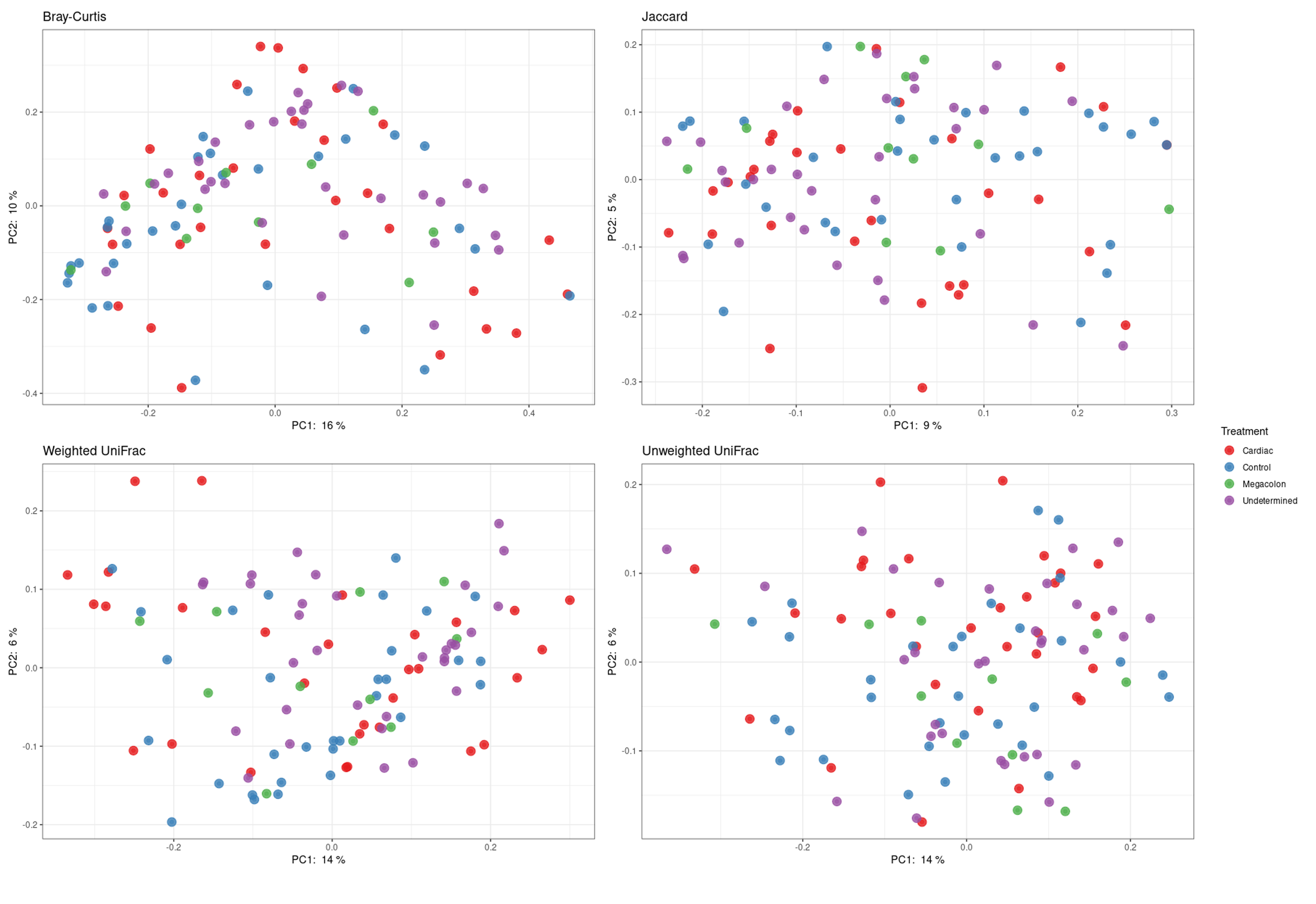

Supplement: Supplementary Figure 1 — Beta diversity plots for non-phylogenetic (Bray-Curtis and Jaccard) and phylogenetic (weighted and unweighted UniFrac) methods in control and the Chagas disease phenotypes (indeterminate, cardiac and megacolon). [file Image_1.TIF]
